# Advancing electrospinning towards the future of biomaterials in biomedical engineering

**DOI:** 10.1093/rb/rbaf034

**Published:** 2025-04-29

**Authors:** Yanjiao Teng, Lin Song, Jie Shi, Qi Lv, Shike Hou, Seeram Ramakrishna

**Affiliations:** Tianjin Key Laboratory of Disaster Medicine Technology, School of Disaster and Emergency Medicine, Tianjin University, Tianjin 300072, China; State Key Laboratory of Pharmaceutical Biotechnology, School of Life Sciences, Nanjing University, Nanjing 210000, China; Tianjin Key Laboratory of Disaster Medicine Technology, School of Disaster and Emergency Medicine, Tianjin University, Tianjin 300072, China; Tianjin Key Laboratory of Disaster Medicine Technology, School of Disaster and Emergency Medicine, Tianjin University, Tianjin 300072, China; Tianjin Key Laboratory of Disaster Medicine Technology, School of Disaster and Emergency Medicine, Tianjin University, Tianjin 300072, China; Department of Mechanical Engineering, National University of Singapore, Singapore 117575, Singapore

**Keywords:** electrospinning, intelligent biomaterials, sustainable biomaterials, healthcare, biomedical application

## Abstract

Biomaterial is a material designed to take a form that can direct, through interactions with living systems, the course of any therapeutic or diagnostic procedure. Growing demand for improved and affordable healthcare treatments and unmet clinical needs seek further advancement of biomaterials. Over the past 25 years, the electrospinning method has been innovated to enhance biomaterials at nanometer and micrometer length scales for diverse healthcare applications. Recent developments include intelligent (smart) biomaterials and sustainable biomaterials. Intelligent materials can sense, adapt to and respond to external stimuli, autonomously adjusting to enhance functionality and performance. Sustainable biomaterials possess several key characteristics, including renewability, a low carbon footprint, circularity, durability, biocompatibility, biodegradability and others. Herein, advances in electrospun biomaterials, encompassing process innovations, working principles and the effects of process variables, are presented succinctly. The potential of electrospun intelligent biomaterials and sustainable biomaterials in specific biomedical applications, including tissue engineering, regenerative medicine, drug delivery systems, brain–computer interfaces, biosensors, personal protective equipment and wearable devices, is explored. More effective healthcare demands further advancements in electrospun biomaterials. In the future, the distinctive characteristics of intelligent biomaterials and sustainable biomaterials, integrated with various emerging technologies (such as AI and data transmission), will enable physicians to conduct remote diagnosis and treatment. This advancement significantly enhances telemedicine capabilities for more accurate disease prediction and management.

## Graphical Abstract

**Figure:**
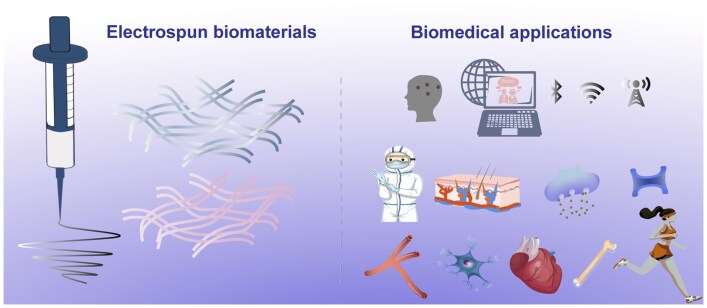

## Introduction

Biomaterial is a material designed to take a form that can direct, through interactions with living systems, the course of any therapeutic or diagnostic procedure [1]. Technological advances have driven innovation in biomaterial fabrication, particularly in nanofiber production techniques, including electrospinning, self-assembly, drawing and centrifugal spinning. Electrospinning is a technique employed to fabricate nanofibers from polymer solutions or melts. This method facilitates the production of exceptionally fine fibers, with diameters ranging from a few nanometers to several micrometers [2]. Compared to traditional fiber structures, electrospun nanofibers have garnered significant attention due to their attributes, including ultra-fine diameters, high surface-to-volume ratios, tunable porosity and superior physical and mechanical properties [3].

**Figure 1.**
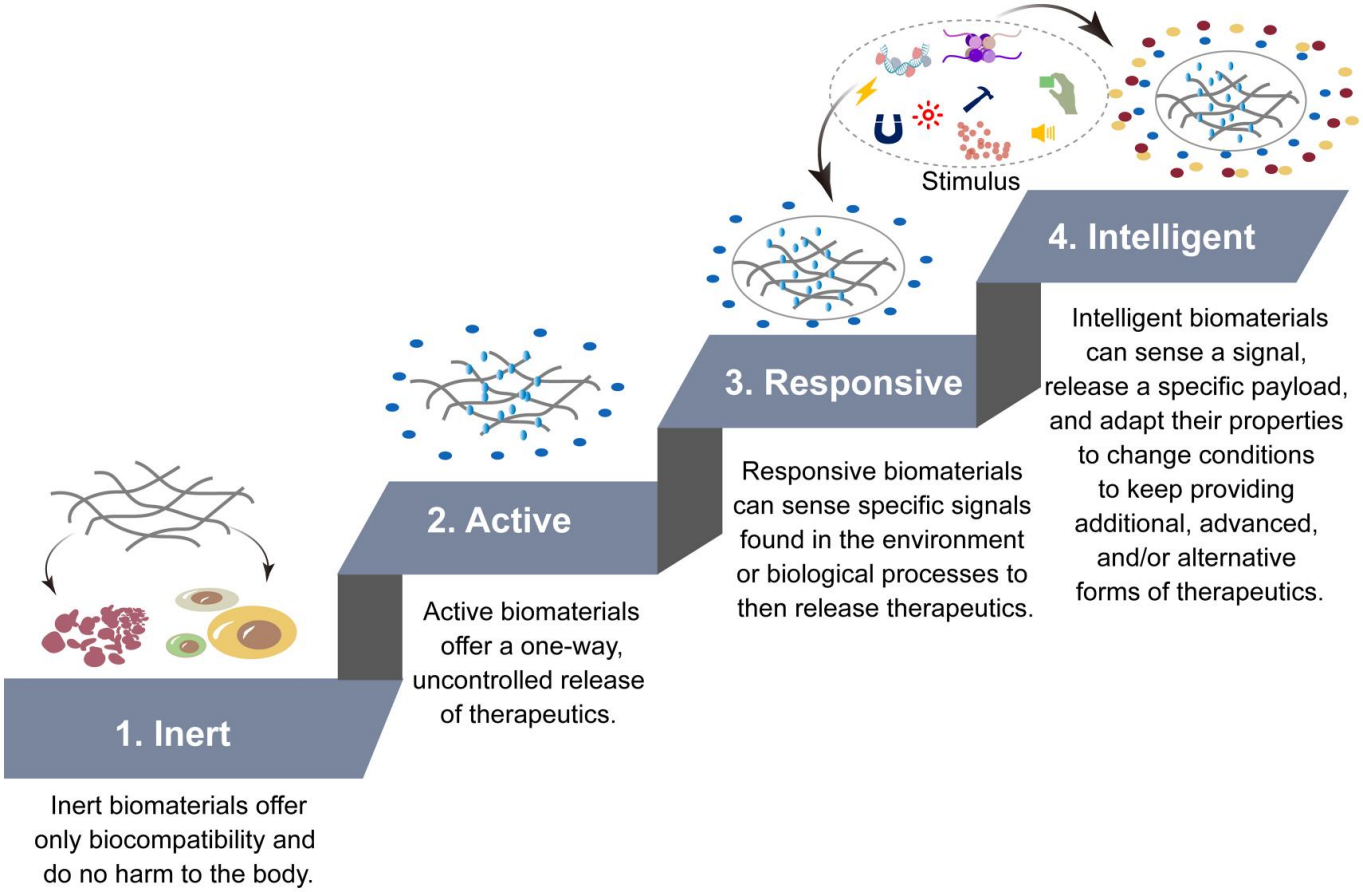
A schematic illustrating the development level of intelligent biomaterials, corresponding to the characteristics of each level. It can be categorized into four levels: inert, active, responsive and intelligent. Some content has been adapted. Reproduced from Ref. [4] with permission of Springer Nature, © 2021.

**Figure 2.**
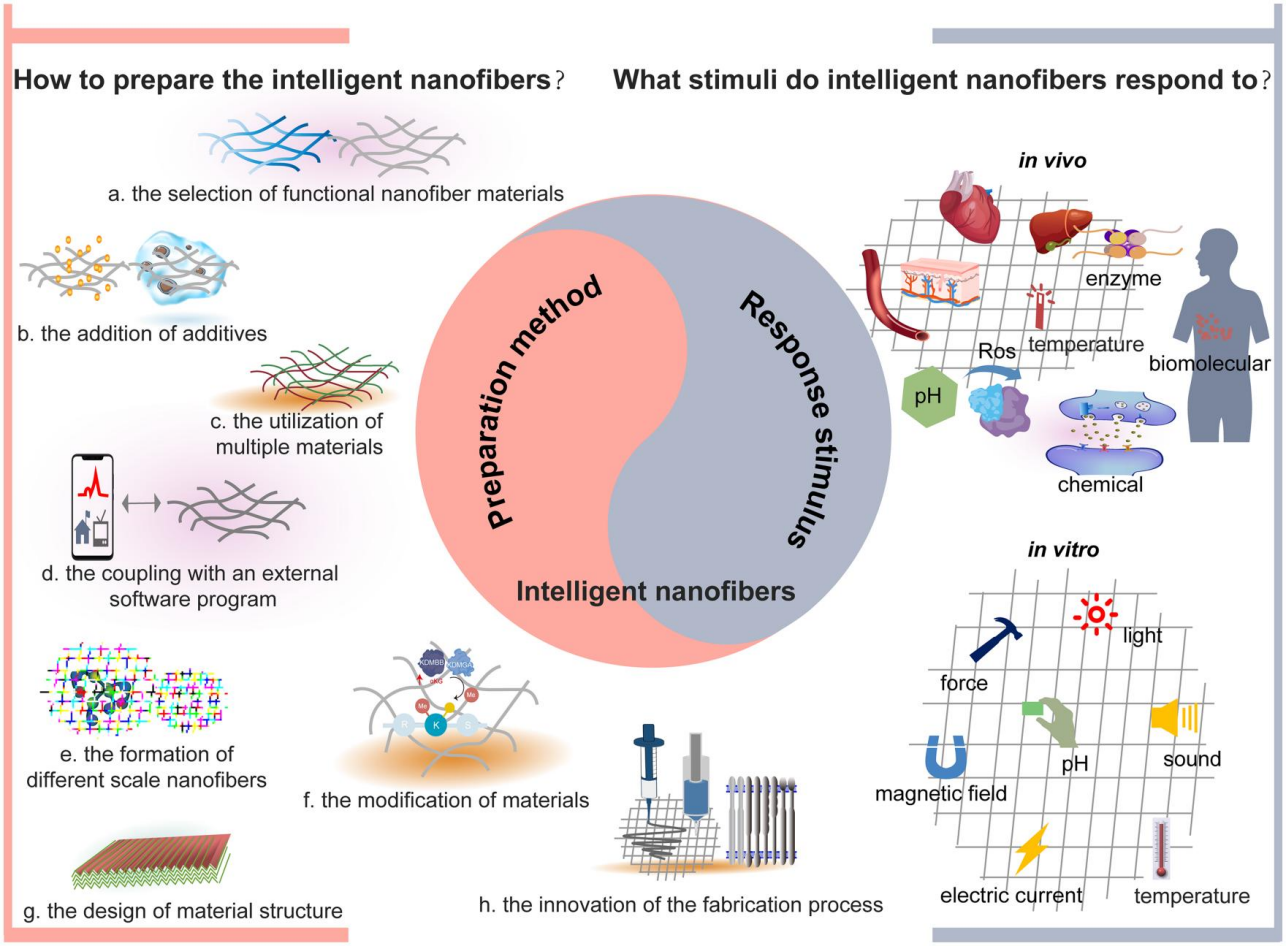
Schematic of the preparation of intelligent nanofibers and the various stimuli they are designed to respond to. Various ways of realizing intelligent nanofibers: (**a**) the selection of functional nanofiber materials; (**b**) the addition of additives; (**c**) the utilization of multiple materials; (**d**) the coupling with an external software program; (**e**) the formation of nano-, micro-, meso- and macro-structuring of nanofibers; (**f**) the modification of materials; (**g**) the design of material structure; (**h**) the innovation of the fabrication process.

Nanofibers are significant in the biomedical areas of intelligent (smart) materials and sustainable materials. The evolution of intelligent biomaterials has progressed through various stages, which can be categorized into four distinct levels: inert, active, responsive and intelligent (Figure 1) [4]. Intelligent materials, can sense, adapt to and respond to external stimuli (e.g. light, temperature, sound, force, electric current, magnetic field, chemicals, pH, enzymes, redox, etc), autonomously adjusting to enhance functionality and performance, like self-repair of tissues, efficient drug release and real-time environmental monitoring [5]. For instance, in the biomedical sector, intelligent nanofibers can release drugs in response to fluctuations in body temperature or pH, providing more precise therapeutic solutions [6]. Intelligent nanofibers can be produced by various methods, as illustrated in Figure 2. They include (a) the selection of functional nanofibrous materials; (b) the addition of additives (e.g. enzymes, cross-linkers, biomolecules, cells); (c) the utilization of multiple materials; (d) the coupling with an external software program; (e) the formation of nano-, micro-, meso- and macro-structuring of nanofibers by a self-assembly method and by using the interaction between molecules (e.g. hydrogen bond, electrostatic force, van der Waals force, etc); (f) the modification of materials; (g) the design of material structure; (h) the innovation of the fabrication process [7, 8].

**Figure 3.**
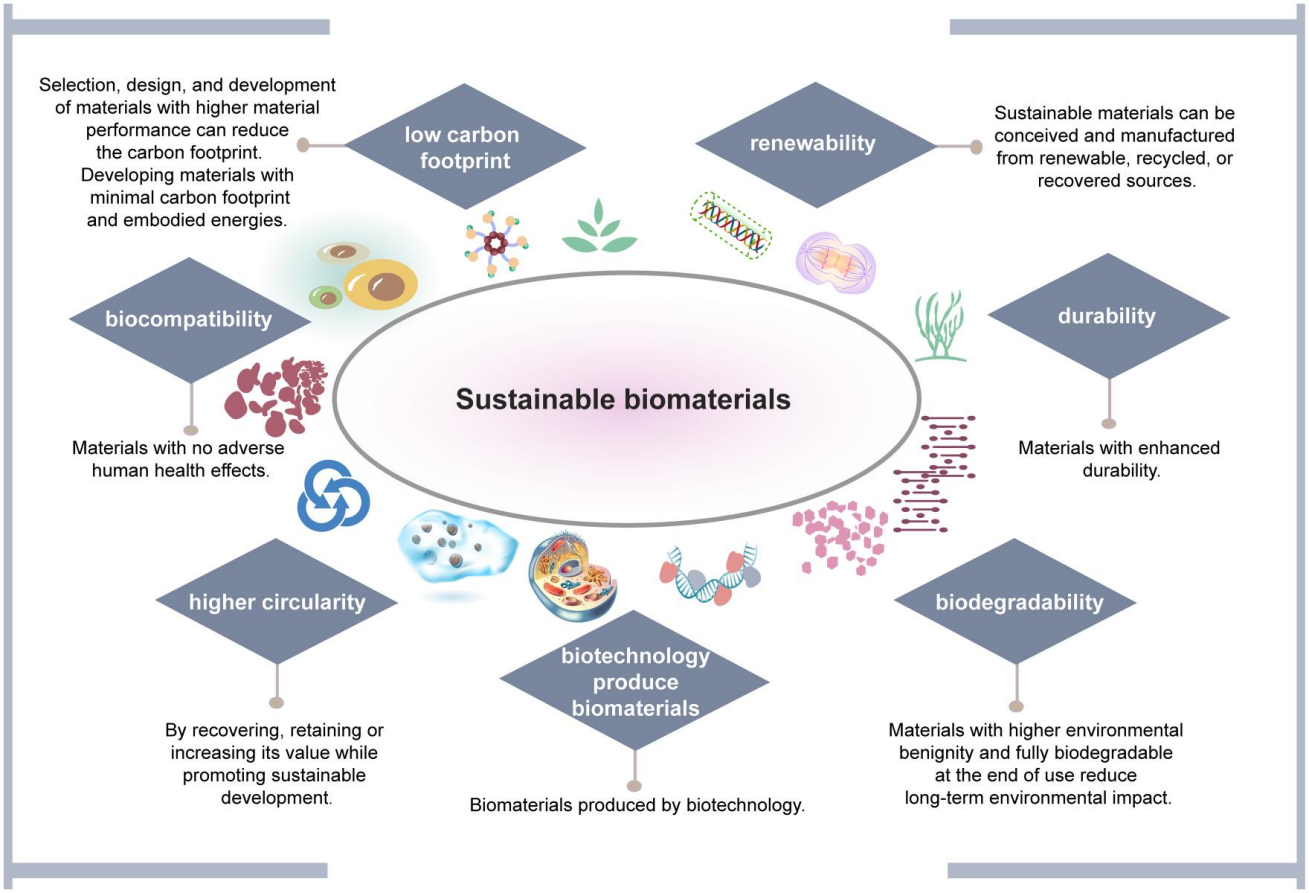
Schematic of the characteristics or attributes of sustainable biomaterials. They include renewability, low carbon footprint, higher circularity, durability, biomaterials produced by biotechnology, biocompatibility and biodegradability.

Generally, sustainable biomaterials possess the following characteristics or attributes (Figure 3): (a) Renewability: sustainable materials can be conceived and manufactured from renewable, recycled or recovered sources. (b) Low carbon footprint: selection, design and development of materials with higher performance and durability. In addition, developing materials with a minimal carbon footprint and low embodied energy [9]. (c) Higher circularity: by recovering, retaining or increasing its value while promoting sustainable development. (d) Durability: materials with enhanced durability. (e) Biomaterials produced by biotechnology: with these technologies, the end-use products can be degraded to be absorbed into the earth system in a shorter period while being least polluting or more environmentally friendly. (f) Biocompatibility: materials that do not pose any adverse effects on human health. (g) Full biodegradability at the end of use: materials with higher environmental benignity and full biodegradability at the end of use reduce long-term environmental impact [9]. In the biomedical field, it is understood that sustainable biomaterials are derived from renewable sources while meeting the biocompatibility and biodegradability requirements of specific biomedical applications. These characteristics enable more effective interactions with human tissues, improving therapeutic outcomes. Furthermore, sustainable biomaterials are associated with reduced immune responses and inflammatory reactions compared to traditional materials. Their degradability mitigates potential risks associated with long-term implantation. Consequently, this decreases the incidence of complications and enhances patient safety and quality of life.

Biosynthesis is the process by which living organisms synthesize biomaterials. This process utilizes enzymes, microenvironments and synthetic biology methods within living organisms to transform artificially constructed modules or precursors into functional biomaterials through chemical reactions, such as self-assembly or polymerization. The synthesis of biomaterials *in vivo* involves non-covalent interactions, covalent bond formation and genetic strategies [10]. For instance, a recent study described a highly efficient Fib-H^EXP^ biosynthesis system for producing substantial quantities of recombinant red fluorescent protein (RFP) from transgenic silkworms. This system also facilitates the functionalization of fibroin-based biomaterials [11]. The application range of biological materials can be further expanded through biosynthesis technology, which provides a pathway to produce sustainable biomaterials.

Herein, we present and discuss the advances and flexibility of the electrospinning technique toward intelligent biomaterials as well as sustainable biomaterials for diverse biomedical applications.

## The electrospinning techniques

### Conventional electrospinning

Standard electrospinning equipment comprises a high-voltage power source, an injection pump, a spinneret equipped with a metal needle and a collector [12]. The high voltage between the metal needle and the collector generates a ‘Taylor cone’ at the needle tip, propelling a charged jet toward the cathode [13]. These nanofibers randomly deposit in a spiral pattern on the collector, forming the desired nanofiber structure [14].

**Figure 4.**
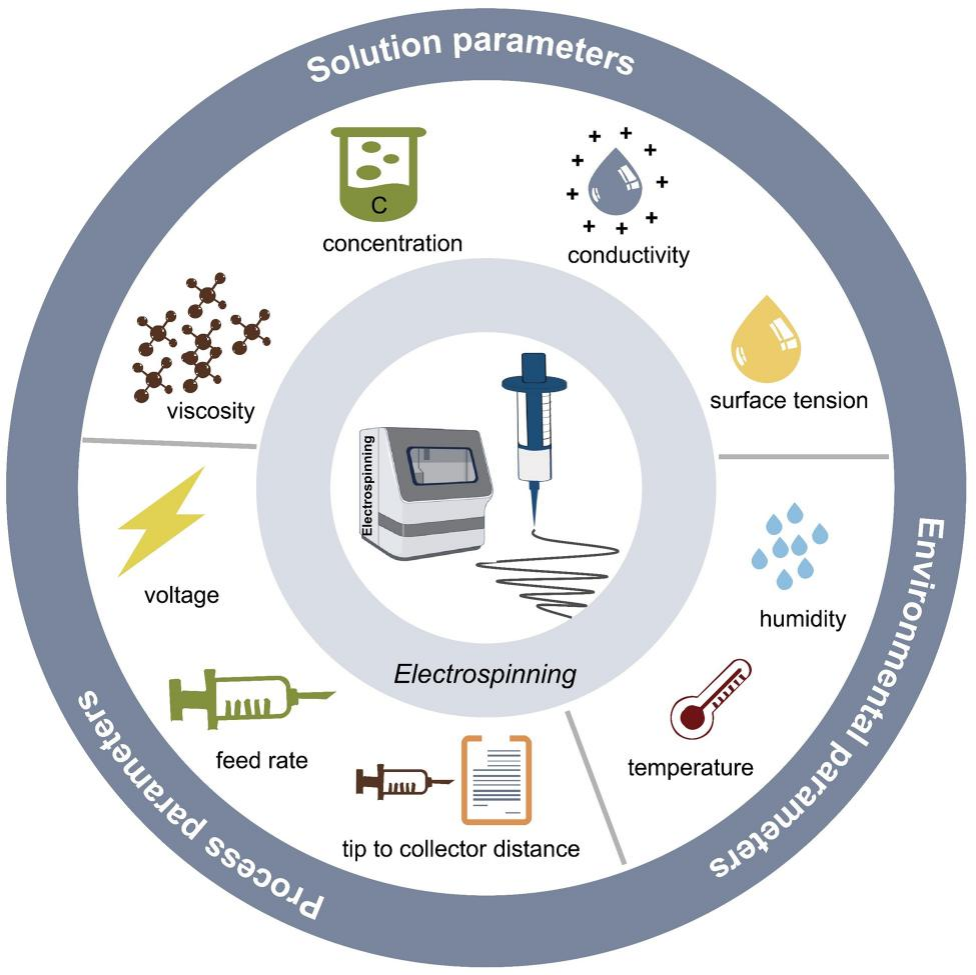
The parameters influencing fiber diameter and morphology in electrospinning mainly include solution, environmental and process parameters.

During the electrospinning process, the size and morphology of the nanofibers can be controlled by adjusting the electrospinning parameters [13]. As illustrated in Figure 4, these parameters can be broadly categorized based on solution (viscosity, concentration, conductivity, surface tension), environmental (temperature, humidity) and process (voltage, feed rate, tip-to-collector distance) parameters [3, 15]. The influence of these parameters on the control of fiber structure is summarized in Table 1. By optimizing these parameters, the controllability and stability of electrospun nanofibrous structures can be significantly enhanced.

**Table 1.** The influence parameters of fiber diameter and morphology in electrospinning

Electrospinning parameters		Fiber diameter	Fiber morphology	Ref.
Solution parameters	Viscosity↑	↑	High viscosity affects solubility and extrusion; low viscosity impacts the formation of continuous fibers	[16]
	Concentration↑	↑	Low concentrations form beaded fibers	[16]
	Conductivity↑	↓	Low conductivity prevents the liquid from stretching; high conductivity leads to incomplete solvent evaporation on the collector	[14]
	Surface tension↑	↑	Large surface tension causes jet instability	[16]
Environmental parameters	Temperature↑	↓	High temperatures can make the fiber diameter distribution uniform	[17]
	Humidity↑	↑	High humidity affects fiber shape; low humidity results in rapid solvent evaporation	[18]
Process parameters	Voltage↑	↓	High voltage can induce an unstable pinning process, resulting in the formation of beads on the fibers	[19]
	Feed rate↑	↑	A high feed rate can result in uneven fiber or bead formation, while a low feed rate may lead to needle clogging	[19]
	Tip to collector distance↑	↓	Small distance, spun fibers tended to adhere	[13]

Based on conventional electrospinning technology, various alternative forms of electrospinning have emerged. These include single-nozzle electrospinning, multi-nozzle electrospinning, coaxial electrospinning, emulsion electrospinning, triaxial electrospinning and melt electrospinning, among others. In Table 2, we analyse the main structures, advantages and disadvantages of these systems.

**Table 2.** Classification and characteristics of electrospinning technology

Electrospinning type	Main structure	Advantages	Disadvantages	Ref.
Single-nozzle electrospinning	Only one nozzle	Simple and widely applied in laboratory-scale studies	Less ideal for sustained drug delivery	[20]
Multi-nozzle electrospinning	Multi-nozzle systems (≥2) spin solutions onto a single collector	Fabrication of diverse compositions of nanofibers, enhance production efficiency, and overcome solvent incompatibility	Electric field interference, nozzle blockage, equipment complexity, and material selection	[21, 22]
Coaxial electrospinning	Core–shell nozzles	Get core–shell structures, hollow fibers, and other specialized morphologies	Fiber morphology is challenging to control, low yield	[23]
Emulsion electrospinning	Emulsion system to make core-shell nanofibers (add surfactants to separate different phases)	Production of functional nanofibers, prevent biomolecular damage, reduce the risk of contamination	Low efficiency	[24, 25]
Triaxial electrospinning	Utilizes three concentrically nested needles to distribute solutions into inner, middle, and outer layers	Achieving controlled drug release	Solvent evaporation and polymer selection	[26]
Melt electrospinning	Heater, air pressure, collector	No harmful solvent residues, lower costs, enhanced safety and eco-friendliness	High polymer melt viscosity, low conductivity and the requirement for strong electric fields	[27]

The raw materials for fabricating electrospun nanofibers include both natural and synthetic polymers, which are widely used in biomedical applications. Table 3 lists common polymers used for electrospinning. Combining natural polymers with synthetic polymers can enhance their spinnability, resulting in fibers with superior biocompatibility and tailored functionalities.

**Table 3.** Examples of electrospun polymers and their characteristics, as well as their biomedical applications

Electrospinning polymers		Characteristic	Biomedical application	Ref.
Natural polymers	Chitosan	Biodegradable, good biocompatibility, antimicrobial, antiviral, hemostatic, minimal cytotoxicity	Medical textiles, wound treatments, wound dressing	[28–30]
	Hyaluronic acid	Moisture, good biocompatibility	Wound dressing	[31]
	Cellulose	Biodegradable, good biocompatibility, good mechanical properties, non-toxic	Water filtration applications, drug delivery	[32, 33]
	Alginate	Good biocompatibility promotes cell growth	Wound treatments	[29]
	Collagen	Good biocompatibility, biodegradable, hemostasis, promote cell growth	Tissue engineering	[34]
	Silk fibroin	Good biocompatibility, biodegradable, good mechanical properties	Angioplasty balloon coating	[35]
	Gelatin	Good biocompatibility, biodegradable, low immunogenicity, low irritability, antigenicity	Medical textiles, tissue engineering	[28, 36]
Synthetic polymers	Ooly(lactic-co-glycolic acid) (PLGA)	Good biocompatibility, good mechanical properties, biodegradable	Tissue engineering	[34]
	Polycaprolactone (PCL)	Good biocompatibility, biodegradable	Tissue engineering	[37]
	Poly(lactic-co-glycolic acid) (PLCL)	Good biocompatibility, biodegradable	Tissue engineering	[38]
	Poly(lactic acid) (PLA)	Environmentally friendly, thermoplastic polymer, good biocompatibility	Tissue engineering, wound dressing	[36, 39]
	Poly(vinyl alcohol) (PVA)	Good biocompatibility, water solubility, film formation	Wound dressing	[30, 31]
	Polyurethane (PU)	Good biocompatibility, non-toxicity, elasticity and good mechanical properties	Tissue engineering	[40]
	Poly(ether urethane) (PEU)	Good mechanical properties, hydrophobic	Tissue engineering	[41]
	Polytetrafluoroethylene (PTFE)	Good thermal and chemical stability, high fracture toughness, good biocompatibility	Tissue engineering	[42]

### Electrohydrodynamic printing

**Figure 5.**
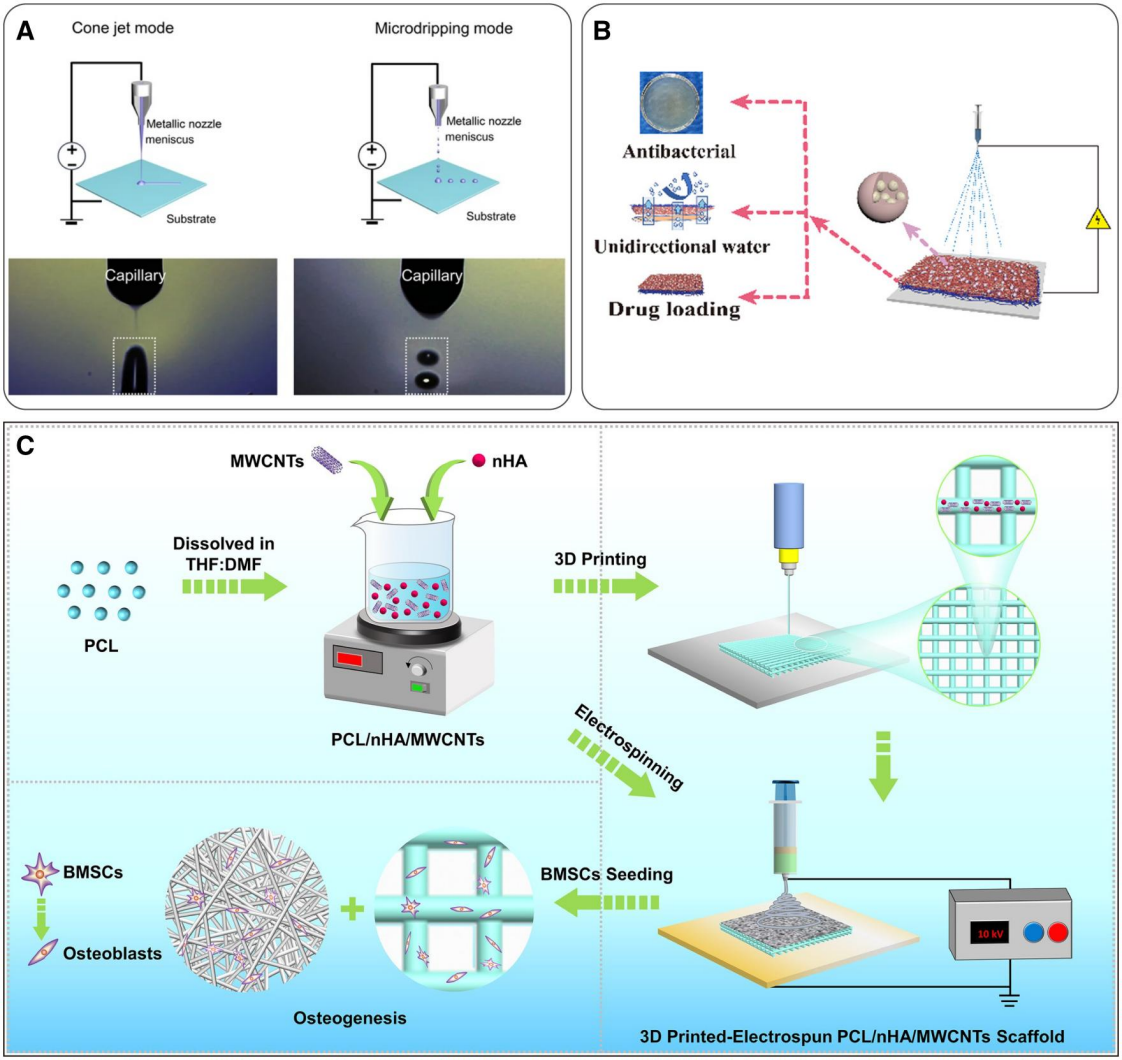
Schematic of several electrospinning strategies. (**A**) Electrohydrodynamic printing enables precise material deposition through specific droplet formation modes, such as microdripping and cone-jet spraying. Reproduced from Ref. [43] with permission of Springer Nature, © 2024. (**B**) Electrospraying involves applying a high voltage to convert the liquid into fine droplets. Reproduced from Ref. [45] with permission of Elsevier, © 2022. (**C**) Electrospinning combined with 3D printing, which can better meet material design needs, supports the development of advanced biomaterials with improved composite structures for practical applications. Reproduced from Ref. [46] with permission of Oxford University Press, © 2022.

Electrohydrodynamic (EHD) printing enables precise material deposition through specific droplet formation modes, such as microdripping and cone-jet spraying [43,44] (Figure 5A). EHD jet printing evolved from electrospinning. The method for feeding materials in solution-based EHD jet printing resembles that used in solution electrospinning [47]. The precision of EHD jet printing is no longer constrained by nozzle diameter, allowing the generation of droplets much smaller than the nozzle size [48]. During the EHD jet printing process, an applied electric field exerts a force on the liquid at the nozzle, influencing its flow and morphology, which leads to the formation of a Taylor cone. This method enables the fabrication of complex nano- and microscale designs, allowing for precise control of fine droplets and surpassing the capabilities of traditional inkjet printers [49].

### Electrospraying

In electrospray technology, a high voltage is applied to convert the liquid into fine droplets [50]. This method is particularly effective for preparing nanoparticles and polymer coatings, as it facilitates the formation of uniform coatings on diverse surfaces (Figure 5B) [45]. Electrospray technology utilizes an electric field to generate a fine mist or droplet spray, one of the electrohydrodynamic atomization (EHDA) technologies [17]. Electrospray technology has been applied to various fields, including drug release, wound healing, capsule and microsphere preparation, and dressings, among others. Electrospinning and electrospray techniques can be utilized in combination. By modulating the viscosity and electric field strength of the solution, it is possible to fabricate both nanofibers and micro/nano particles using one device.

### Electrospinning + 3D printing

3D printing, commonly referred to as additive manufacturing, is an advanced rapid prototyping technology that enables the reliable and efficient creation of three-dimensional objects. As a bottom-up manufacturing approach, 3D printing incrementally fulfills design specifications by layering materials [51]. This additive manufacturing technique allows for producing items with specific three-dimensional geometries, significantly reducing design costs and operational complexities. Combining 3D printing with electrospinning could better meet material design needs, supporting the development of advanced biomaterials with improved composite structures for practical applications (Figure 5C) [46]. The combination method encompasses various approaches, including electrospinning onto 3D-printed scaffolds, 3D printing onto electrospun fibers, alternating use of 3D printing and electrospinning, decorating or infusing 3D-printed scaffolds with electrospun fibers, fabricating electrospun scaffolds on 3D-printed collectors or templates and others [52].

## Application of electrospun biomaterials

### Tissue engineering and regenerative medicine

**Figure 6.**
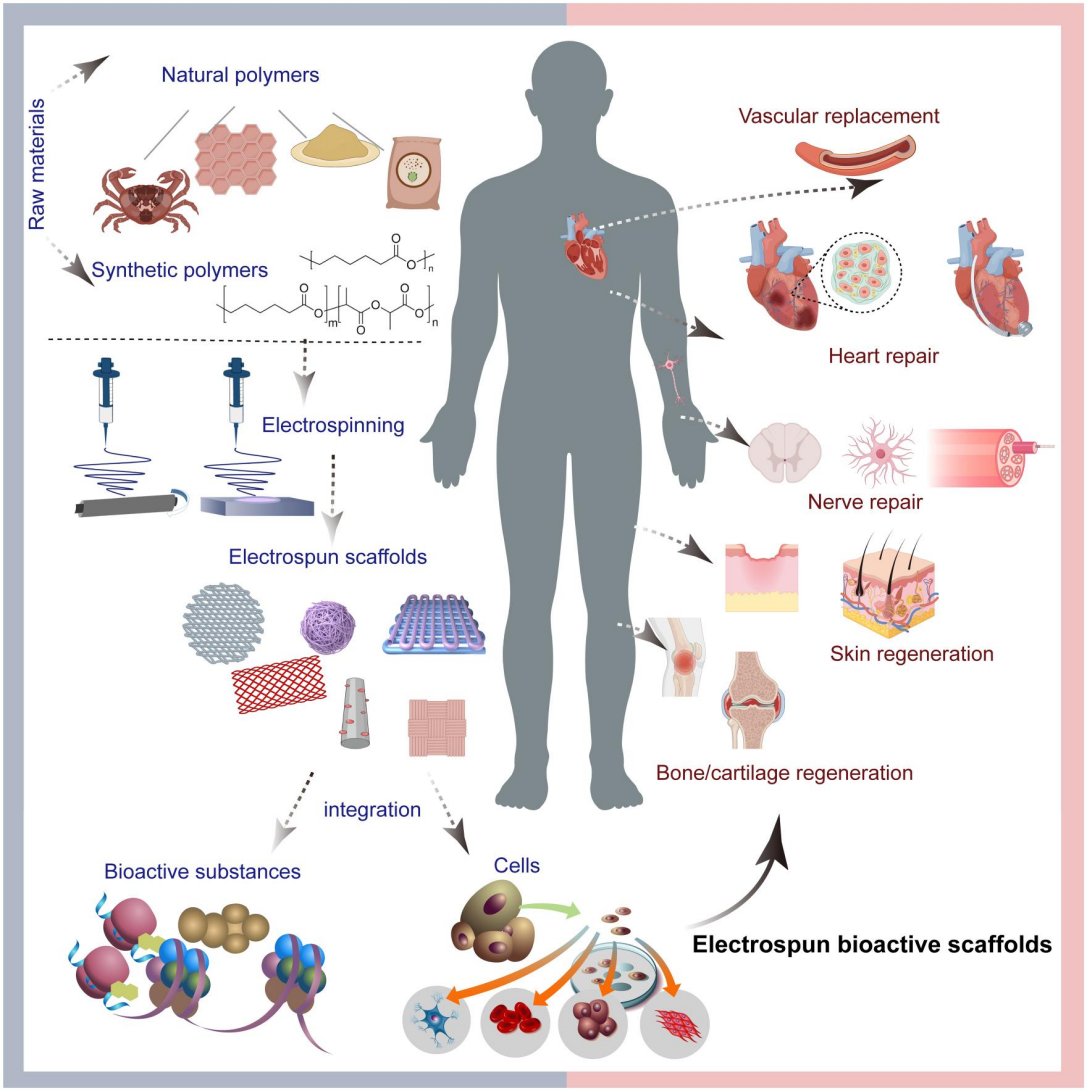
Schematic of electrospun biomaterial scaffolds applied in tissue engineering and regenerative medicine (e.g. vascular replacement, heart repair, nerve repair, skin regeneration, bone/cartilage regeneration).

Electrospun nanofibers exhibit significant potential in replicating ECM structures in tissue engineering scaffolds, as they can closely mimic the natural ECM, thereby facilitating cell attachment, growth and tissue regeneration [53]. Electrospun scaffolds are widely used in tissue engineering and regenerative medicine (Figure 6).

#### Heart repair

**Figure 7.**
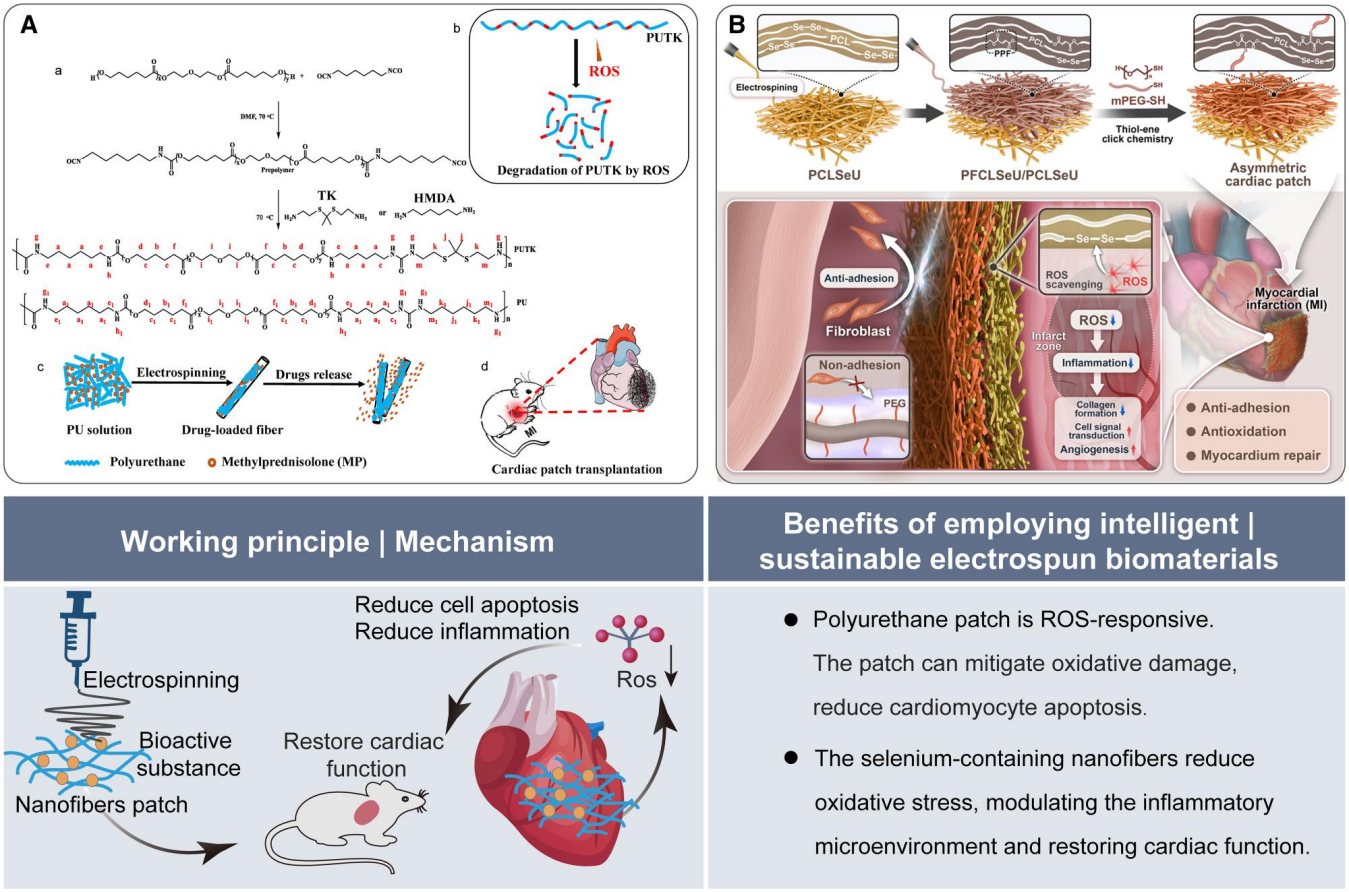
The application of electrospun nanofibers for heart repair. (**A**) Fibrous patch for treating MI. (**a**, **b**) The synthesis and characterization of the material. (**c**) Fabrication of methylprednisolone (MP)-loaded electrospun patches. (**d**) Implanting a cardiac patch on MI surfaces to regenerate damaged heart tissue. Reproduced from Ref. [56] with permission of Elsevier, © 2019. (**B**) A dual-layer nanofibrous membrane exhibiting anti-oxidation properties to enhance cardiac repair following MI *in vivo*. Reproduced from Ref. [57] with permission of Elsevier, © 2024.

Cardiovascular diseases (CVDs) pose a substantial threat to human health [54]. Electrospun nanofibers possess the potential to simulate the structure of cardiac tissue; their superior mechanical properties can enhance cardiomyocyte function and improve biocompatibility [55]. Scaffolds composed of intelligent biomaterials play a crucial role in heart repair. A recent study presented a reactive oxygen species (ROS)-responsive degradable elastic polyurethane (PUTK) fiber patch loaded with methylprednisolone (MP) for treating myocardial infarction (MI) in rats. ROS-responsive PUTK responds to ROS through specific functional groups within its molecular structure, namely the thioacetal bond. This interaction facilitates degradation and the release of bioactive components, thereby mitigating oxidative damage and reducing cardiomyocyte apoptosis (Figure 7A) [56]. In another study, nanofibrous patches were developed by electrospinning a combination of selenium polyurethane (PU) and polyethylene glycol (PEG). The selenium-containing nanofibers demonstrated a capacity to reduce oxidative stress, thereby modulating the inflammatory microenvironment and effectively restoring cardiac structure and function (Figure 7B) [57].

#### Vascular replacement

Vascular grafts made from sustainable biomaterials offer distinct advantages in managing CVDs [38]. Owing to the good degradability of the material, these grafts can be resorbed after fulfilling their function of supporting blood vessels. This property effectively mitigates inflammatory reactions and other complications associated with grafts made from non-degradable materials. Furthermore, the grafts facilitate tissue remodeling and promote vascular regeneration during degradation. Electrospun vascular grafts feature highly interconnected nanofibers that replicate the structure of the natural extracellular matrix (ECM) [58]. By altering the material's composition and the fibers' diameter, orientation and arrangement, it is possible to adjust the degradability and mechanical properties. Wu *et al.* [59] prepared vascular grafts based on poly(L-lactide-co-e-caprolactone) (P(LLA-CL)), chitosan and collagen utilizing bidirectional gradient electrospinning. The incorporation of biodegradable biomaterials enhanced the degradation of vascular grafts while exhibiting favorable biocompatibility.

#### Nerve repair

**Figure 8.**
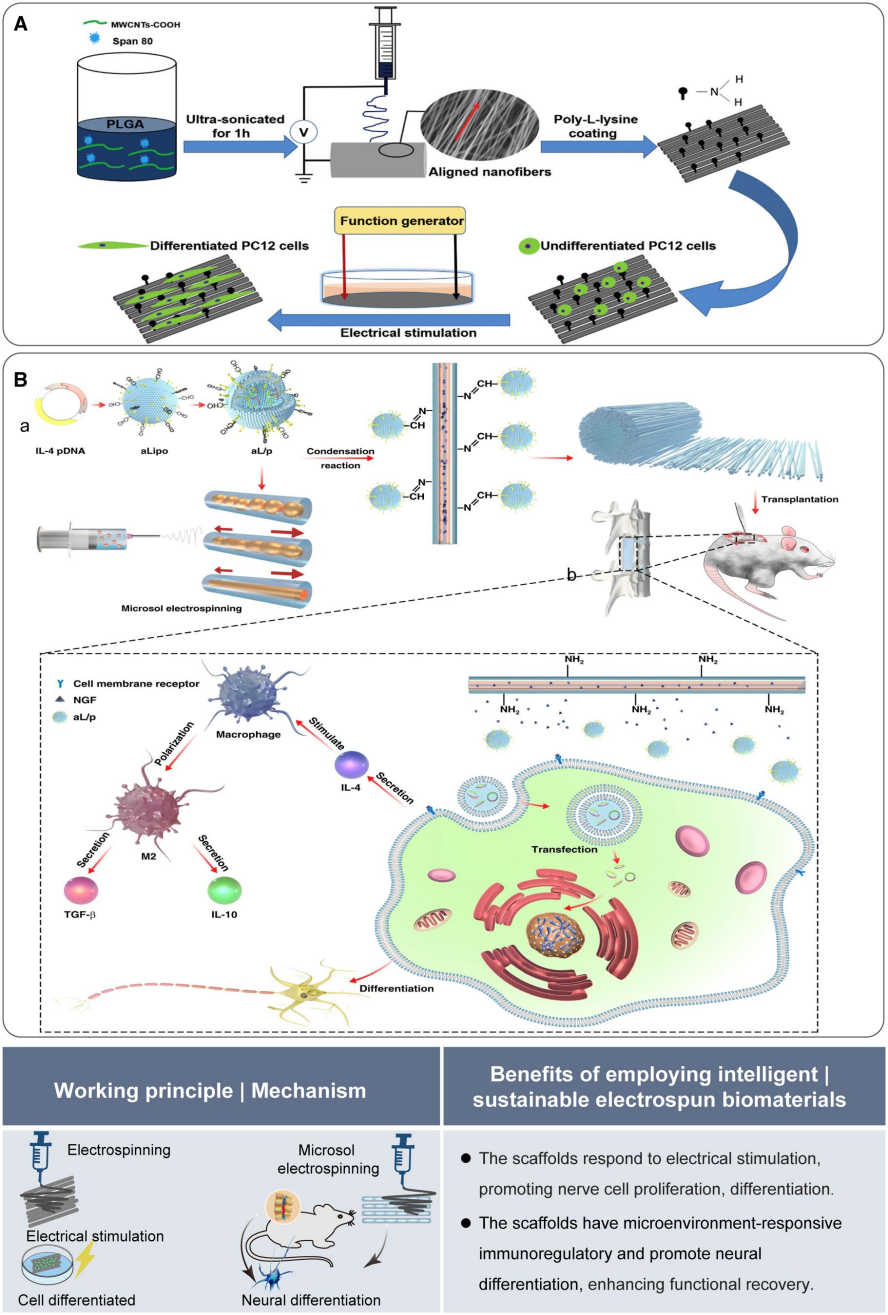
The application of electrospun biomaterials for nerve repair. (**A**) Schematic of MWCNTs scaffold fabrication showing that scaffolds can promote PC12 cell proliferation and differentiation. Reproduced from Ref. [60] with permission of Elsevier, © 2018. (**B**) Schematic of scaffold construction for spinal cord injury treatment, featuring immune modulation and nerve regeneration in response to the microenvironment. Reproduced from Ref. [61] with permission of Springer Nature, © 2021.

The limited self-healing and functional recovery capacity of nerves is a significant challenge in modern medicine [2]. Nanofibrous materials can improve the growth state of nerve cells through electrical stimulation, which reflects the adaptability and responsiveness of intelligent biomaterials. Wang *et al.* [60] conducted a study to investigate the response of nerve cells to an aligned nanofibrous scaffold composed of PLGA and multi-walled carbon nanotubes (MWCNTs). Their findings indicated that when integrated with electrical stimulation, the scaffold allows for an effective response to such stimulation. This response promotes nerve cell proliferation, differentiation, neurite extension and myelination, thereby highlighting its potential applications in nerve regeneration (Figure 8A). Responding to alterations in the microenvironment *in vivo* is another way intelligent biomaterials can react to stimuli. In another study, it was reported that a core–shell structure could sustain the release of the nerve growth factor (NGF), respond to microenvironmental immune regulation, promote neural differentiation and enhance functional recovery (Figure 8B) [61].

#### Bone regeneration

**Figure 9.**
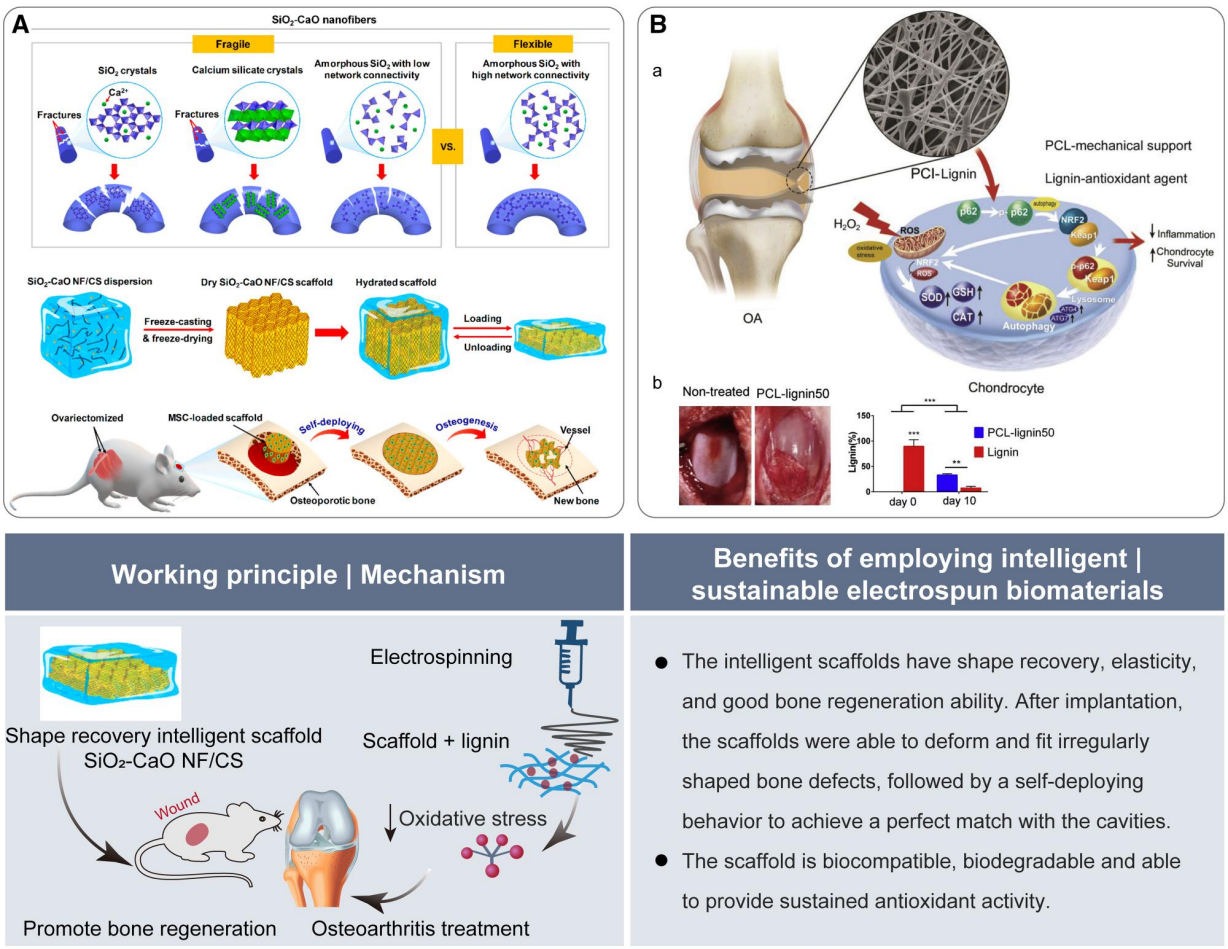
The application of electrospun biomaterials for bone regeneration. (**A**) Schematic of preparing a smart elastic nanofiber-based 3D scaffold to improve rat bone repair. Reproduced from Ref. [62] with permission of American Chemical Society, © 2019. (**B**) Schematic of PCL-lignin nanofibers membrane for osteoarthritis treatment and effect of scaffolds on inhibiting osteoarthritis *in vivo*. Reproduced from Ref. [63] with permission of Elsevier, © 2019.

Nanofibrous membranes produced through electrospinning technology closely mimic the composition and architecture of ECM fibers. This resemblance provides essential structural support that promotes the ingrowth of bone cells. The application of intelligent biomaterials for bone tissue repair is on the rise. In a recent study, it was reported that the achievement of brittleness transformation in bioactive SiO_2_–CaO glass nanofibers was achieved through the regulation of crystallization and chain conformation. This process was combined with a shape-recovery chitosan layer to prepare SiO_2_–CaO NF/CS smart scaffolds. The smart scaffolds exhibited shape recovery, elasticity and a good ability for bone regeneration, providing a new solution for repairing complex bone defects (Figure 9A) [62]. Excessive levels of free radicals, particularly ROS, are a significant contributor to the degradation of cartilage. Lignin is an antioxidant, and the polyphenolic hydroxyl groups in lignin can effectively neutralize free radicals. Liang *et al.* [63] proposed PCL and PCL-grafted lignin (PCL-g-lignin) copolymer. The scaffold was responsive to oxidative stress when stimulated by H_2_O_2_, and it was both biocompatible and biodegradable. This makes it an effective material for treating osteoarthritis (Figure 9B).

#### Skin regeneration

**Figure 10.**
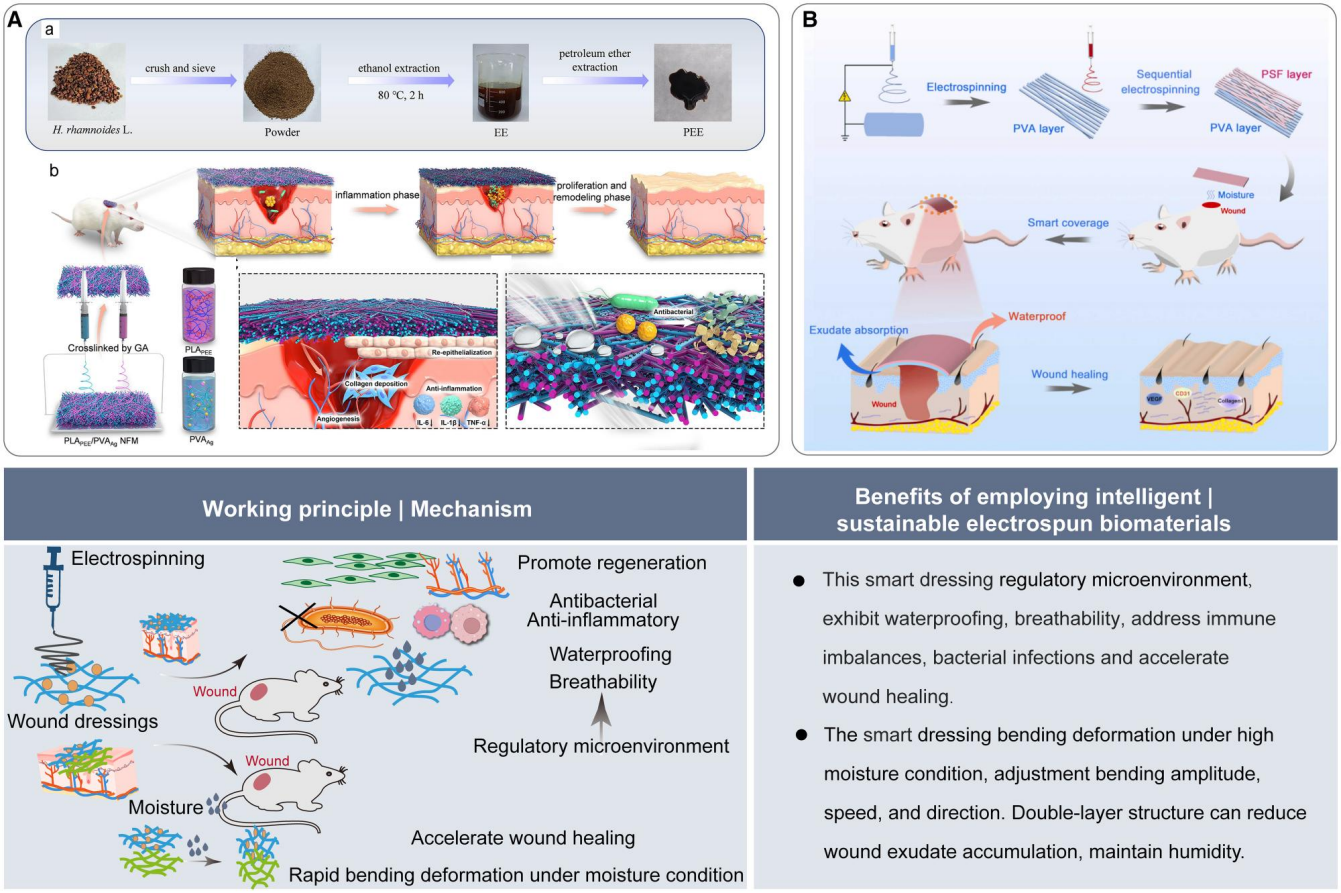
The application of electrospun biomaterials for skin regeneration. (**A**) The schematic of electrospun smart dressing illustrates its role in enhancing wound healing. (**a**) Natural-plant drugs extraction from *H. rhamnoides* L. (**b**) Wound dressing with functionality for accelerating wound healing. Reproduced from Ref. [22] with permission of Elsevier, © 2024. (**B**) The schematic of bioinspired smart dressing fabrication by electrospinning and the promoted healing effect. Reproduced from Ref. [66] with permission of Elsevier, © 2024.

Electrospun nanofibrous membranes exhibit flexibility and can be tailored to conform to the shapes of skin wounds. Their high porosity and exceptional pore interconnectivity are essential for effective exudate drainage and gas exchange [64]. Intelligent dressings represent a crucial element in the field of skin tissue engineering. These dressings can respond appropriately to the wound site based on material properties, enabling real-time monitoring of wound conditions and environmental changes [65]. Xu *et al.* [22] developed a bioactive electrospun smart dressing with anti-inflammatory and antibacterial properties. This smart dressing demonstrated waterproofing, breathability and liquid absorption capabilities, which significantly accelerated wound healing (Figure 10A). In another study, a smart dressing composed of polyvinyl alcohol/polysulfone (PVA/PSF) was reported, inspired by the microscopic structure of pine cones and their hyperelastic deformation characteristics. This biomimetic smart dressing demonstrated rapid deformation under high humidity conditions and could readily adjust its bending amplitude, speed and direction to conform to the irregular contours of human wounds. Smart dressings effectively reduced the accumulation of exudate in wounds while preserving optimal humidity levels. Concurrently, the PSF layer is highly hydrophobic and acts as a barrier against various aqueous solutions, preventing external contaminants from entering the wound site and promoting accelerated wound healing (Figure 10B) [66].

Recent investigations into intelligent dressings are grounded in using flexible electronic materials. A fully integrated, battery-free, wireless, intelligent wound dressing has been developed using flexible electronic technology for wound hemostasis, healing, drug release and other detection purposes [67]. The collection, transmission and processing of information can be controlled by miniaturized circuits and smartphones. In a recent study, a wireless, battery-free, intelligent wound dressing was developed and integrated with a drug delivery module. This dressing was designed as a double-layer patch that monitored wound temperature, pH and uric acid levels, enabling real-time detection of bacterial infections. Additionally, it facilitated the electrically controlled delivery of antibiotics for effective treatment of infections [6]. The development of electrospun dressings is expected to integrate flexible electronic materials, miniaturized circuits and smartphone systems to treat wounds and monitor them in real-time.

### Drug delivery systems

**Figure 11.**
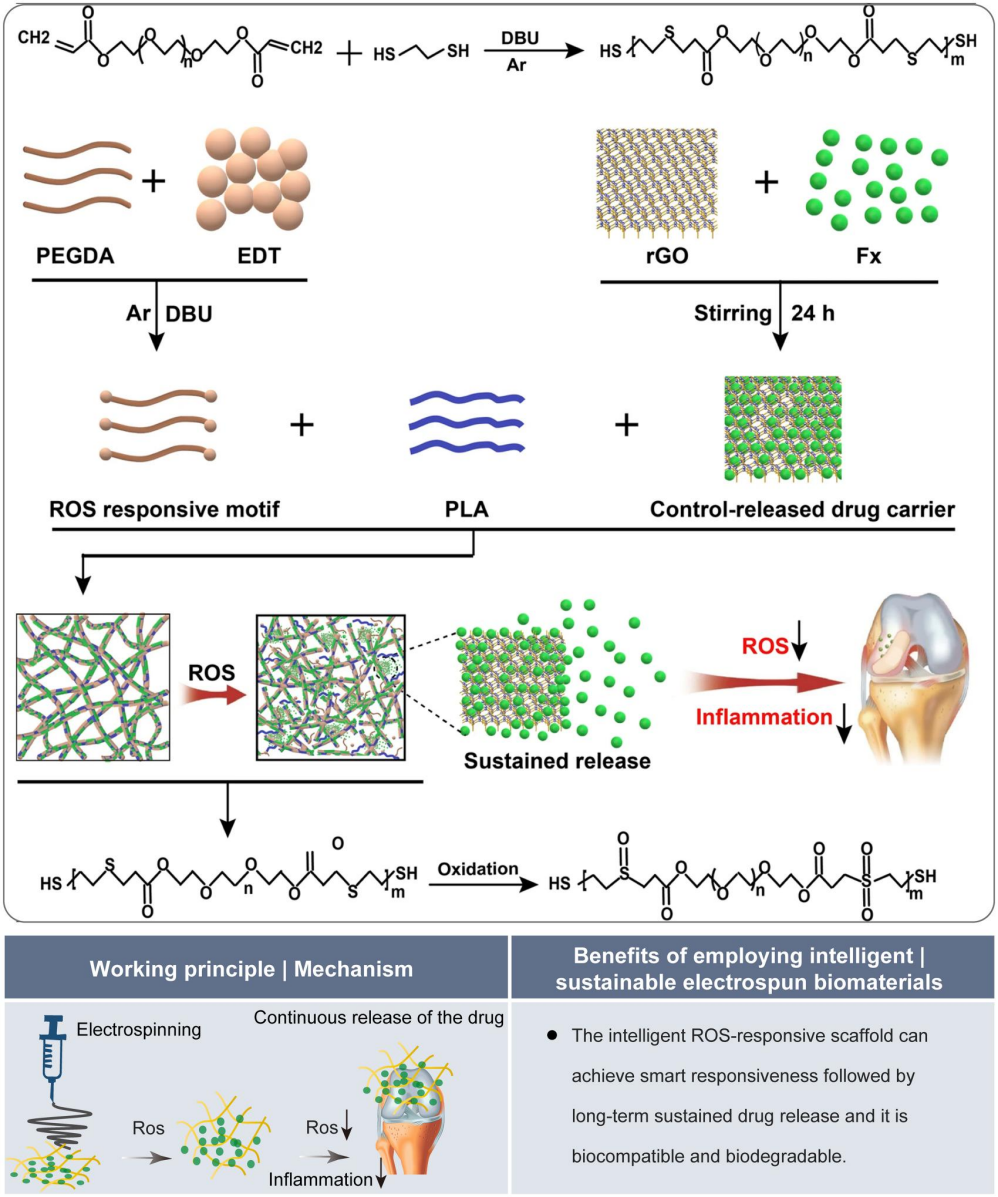
The application of electrospun biomaterials for drug delivery. A schematic illustration of the PPGF nanofiber membrane, designed as a ROS-responsive drug delivery system for the sustained release of Fx in the treatment of osteoarthritis. Reproduced from Ref. [68] with permission of Springer Nature, © 2022.

Electrospun nanofibers offer several advantages in intelligent drug delivery systems, including effective drug adsorption and tunable drug release. They can facilitate the intelligent regulation of drug release through both endogenous and external stimuli. Under endogenous stimuli (e.g. temperature, pH, enzyme activity, ROS levels and others), electrospun fibers can intelligently modulate drug release by responding to changes in physiological and pathological microenvironments. Similarly, electrospun fibers can achieve intelligent control of drug release by reacting to these external factors under exogenous stimuli (e.g. light, electricity, magnetic fields, sound and more). For instance, a recent study introduced a ROS-responsive nanofibrous membrane, which was composed of PLA, polyethylene glycol diacrylate with ethylenediamine tetraacetic acid (PEGDA-EDT) and reduced graphene oxide (rGO) loaded with fucoxanthin (Fx). This innovative material served as a drug delivery system for treating osteoarthritis [68]. The ROS responsiveness of PPGF nanofibrous membranes is achieved by introducing PEGDA-EDT as the ROS response group. The hydrophobic thioether groups present in PEGDA-EDT undergo rapid oxidation to form sulfone or sulfoxide groups when exposed to ROS (e.g. hydrogen peroxide). This oxidation process induced structural changes within the polymer chain, thereby facilitating the release of drugs. In addition, the oxidized PEGDA-EDT also exhibited hydrophilicity, further accelerating the degradation of the fiber film and thus achieving continuous drug release (Figure 11).

### Biosensors

Biosensors are increasingly recognized for their pivotal role in the biomedical field. The electrospun nanofibrous structure significantly enhances the surface area, improves binding efficiency and reaction rates and creates optimal conditions for biosensor applications. In the context of disease diagnosis, biosensors can identify critical biomarkers (e.g. proteins and nucleic acids), facilitating the early detection of chronic diseases, including cancer and cardiovascular disorders [69]. Furthermore, they can monitor vital signs such as blood pressure, blood glucose levels and blood oxygen saturation to support real-time health surveillance [60]. Additionally, biosensors play a pivotal role in precision drug delivery within the field of oncology. They facilitate the targeted administration of therapeutics to lesion sites, thereby enhancing therapeutic efficacy while minimizing adverse side effects [70]. This underscores their significant value in personalized medicine. The advancement of these technologies not only enhances the efficiency of medical services but also provides patients with more convenient and personalized health management solutions.

In the forthcoming period, it is anticipated that there will be a significant increase in the application of biomaterials for sensors designed to facilitate noninvasive measurement of blood gases (e.g. CO, SO_2_, CO_2_) and vital signs. This advancement has the potential to minimize infection risks and complications while enabling continuous monitoring of patient conditions.

### Wearable devices

Wearable devices are emerging as the next breakthrough in fitness, personal health monitoring and public health. Numerous wearable devices, such as earbuds, smartwatches and wristbands, are available on the market. They establish connections with smartphones or remote smart terminals via Bluetooth or wireless networks, facilitating real-time data transmission for monitoring vital signs, including heart rate, pulse, sleep quality and step count [71]. Nanofibrous materials are lightweight, soft and well-suited to body movements. Their ultrathin and flexible networks conform to complex skin deformations and adhere closely to the skin surface, enabling the precise detection of subtle signals that play a transformative role in wearable sensors [72].

**Figure 12.**
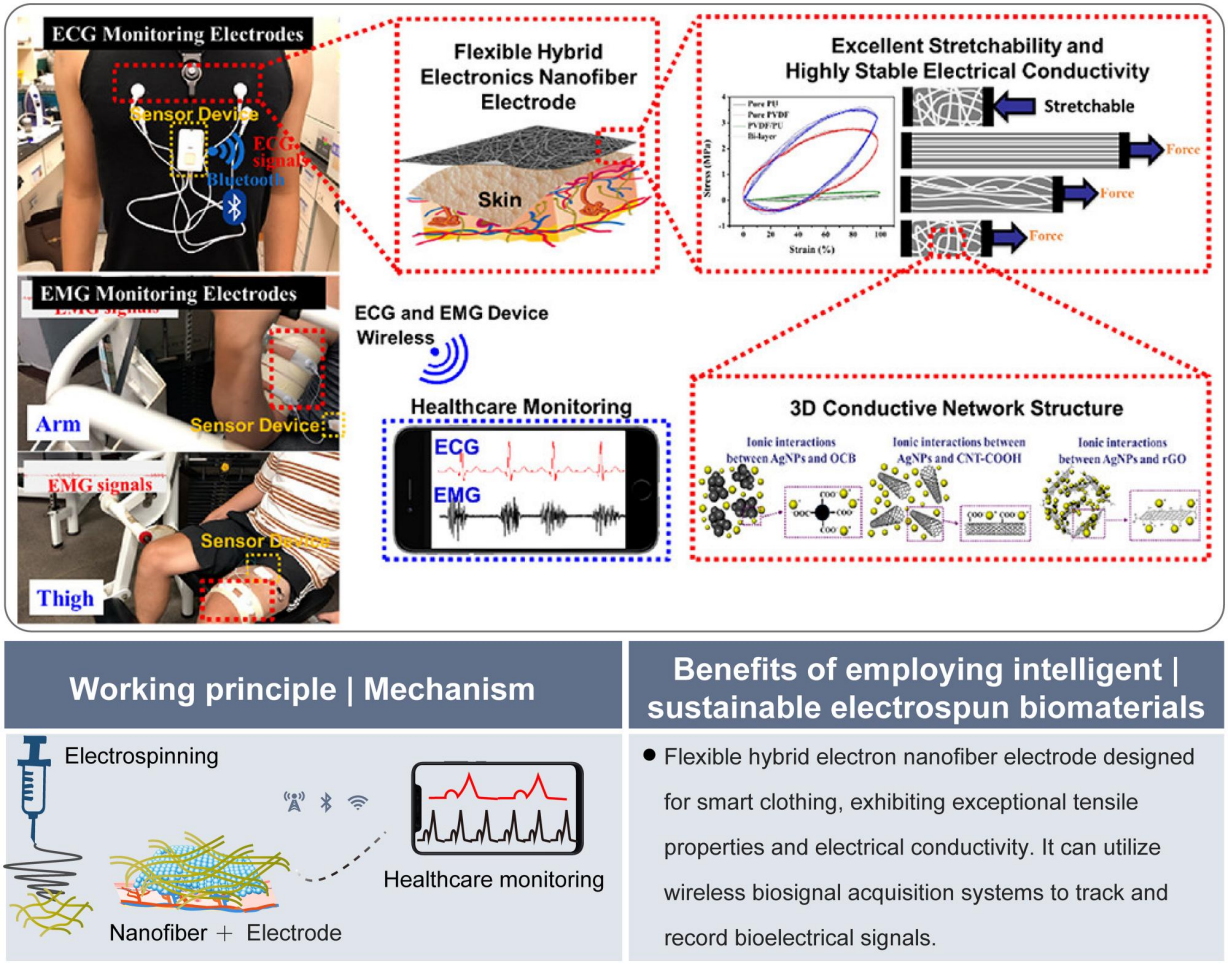
The application of electrospun biomaterials for wearable devices. The flexible hybrid electron nanofiber electrode exhibits excellent stretchability and highly stable electrical conductivity, making it effectively applicable in smart clothing. Reproduced from Ref. [74] with permission of American Chemical Society, © 2022.

Wearable devices are expected to integrate a diverse array of sensors, flexible electronics, nanofibrous biomaterials and big data analytics. This integration aims to achieve multifunctional detection across various human physiological parameters. Consequently, it is anticipated to facilitate personalized health monitoring, enable early detection of health risks and significantly enhance the management of chronic diseases [69]. A recent study presented a muscle fiber-inspired electrospun nanostructure-enhanced conductive fiber (ERCF) designed for intelligent wearable optoelectronics and energy generation applications. The ERCF was produced through electrospinning technology, coupled with the *in situ* formation of silver nanoparticles (AgNPs). This innovative material demonstrated exceptional strain-insensitive conductivity, mechanical strength and resilience, making it suitable for smart gloves and other wearable device applications [73]. In another study, a flexible hybrid electron nanofibrous electrode designed for smart clothing demonstrated exceptional tensile properties and highly stable electrical conductivity. It could reliably track and record bioelectrical signals such as electromyograms (EMG) and electrocardiograms (ECG), by utilizing wireless biosignal acquisition systems (Figure 12) [74].

In wearable devices, biodegradable sensor biomaterials are pivotal for continuous health monitoring and enhancing in-body sensing capabilities. Most currently employed sensors are used externally or noninvasively but necessitate surgical removal after use, subjecting patients to potential complications. In the future, the sensors constructed from sustainable biomaterials have the potential to significantly alleviate these issues by enhancing patient comfort. They minimize foreign body reactions associated with implants while concurrently reducing medical waste due to their inherent degradability.

### Personal protective equipment

Despite technological advancements, viral infections continue to profoundly impact humanity, causing increased global morbidity, mortality and substantial economic losses. Millions of people have lost their lives in the COVID-19 pandemic since 2019. This highlights the critical need to develop novel antiviral personal protective equipment (PPE) with robust antibacterial and antiviral properties.

**Figure 13.**
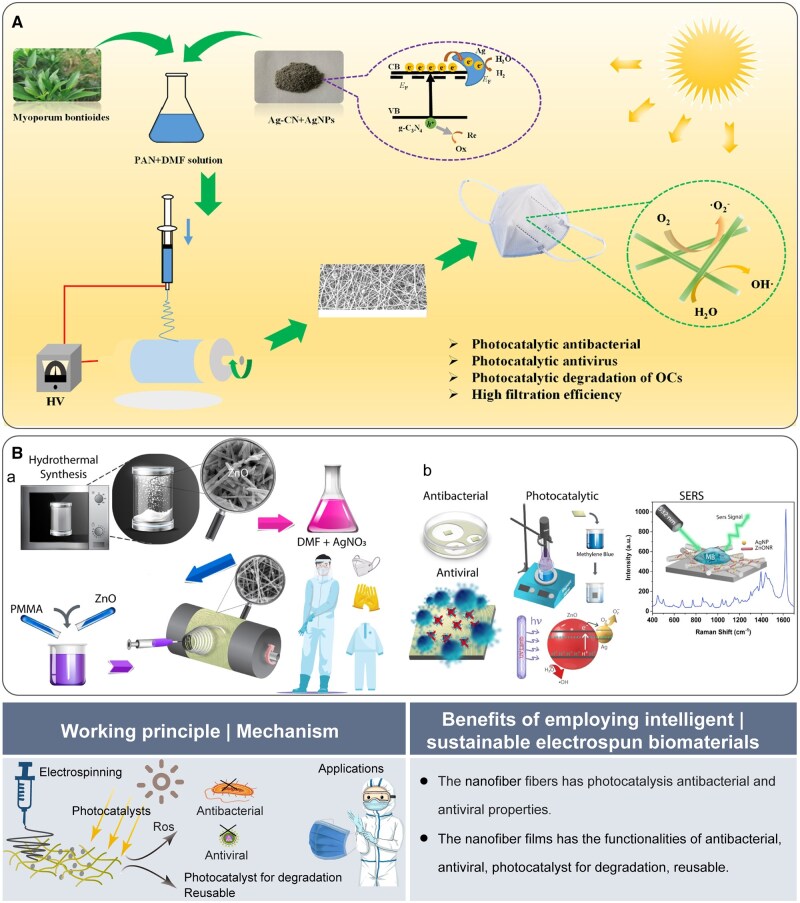
The application of electrospun biomaterials for personal protective equipment. (**A**) Schematic of PAN/M nanofibrous membrane designed and fabricated to serve as the mask filter layer, offering exceptional airflow permeability and high particle filtration efficiency. Reproduced from Ref. [75] with permission of Elsevier, © 2022. (**B**) Fabrication of NFs. (**a**) The NFs were fabricated via electrospinning and utilized to produce protective clothing. (**b**) Multifunctional performance test of NFs. Reproduced from Ref. [76] with permission of American Chemical Society, © 2021.

Electrospun nanofibers can achieve antibacterial functionality through surface treatment or incorporation of antimicrobial agents, with various applications in the biomedical field and household textiles. Similarly, PPE made from fiber materials will integrate intelligent technologies that include sensors responsive to environmental changes. These fibers can modify their structure or release antibacterial and antiviral agents upon contact with viruses or harmful substances after exposure to light, thereby enhancing their protective efficacy. Chen *et al.* [75] designed a reusable nanofibrous membrane consisting of polyacrylonitrile (PAN) combined with silver/graphite carbon nitride (Ag-CN), plant extract and silver nanoparticles (NPs). This membrane exhibited photocatalytic antibacterial and antiviral properties through the electrospinning process and was intended for use in medical masks. When exposed to light, Ag-CN can utilize light energy to produce ROS, which kills pathogens on the mask surface, thereby giving the mask the potential to be reused (Figure 13A). In another study, multifunctional nanofibrous films (NFs) were prepared, exhibiting antibacterial, antiviral and photocatalytic properties that enable the degradation of organic pollutants, thereby providing self-cleaning and rapid sensing capabilities. These photoresponsive nanofibers (NFs) have high potential for protective clothing applications (Figure 13B) [76].

### Brain–computer interface

**Figure 14.**
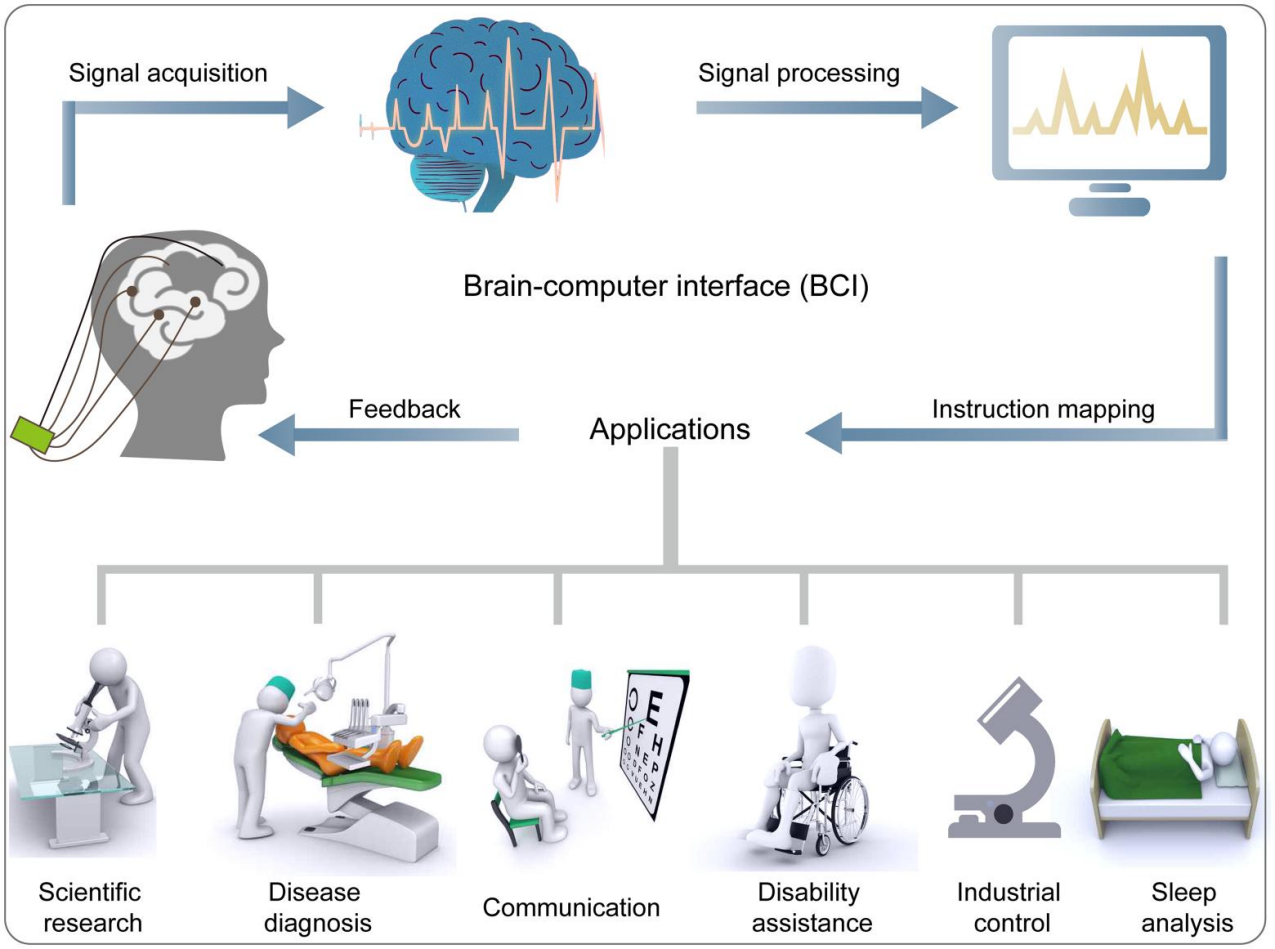
The schematics and applications of BCI. They include scientific research, disease diagnosis, communication, disability assistance, industrial control and sleep analysis. Some content has been adapted. Reproduced from Ref. [77] with permission of American Chemical Society, © 2023.

Brain–computer interface (BCI) facilitates direct interaction between the brain and the external environment through neural interfaces. They offer diverse functions, including monitoring, protection, enhancement and recovery. Specifically, BCI can replace or restore natural output that may be lost due to injury or illness by transmitting signals. This technology provides a vital means of communication for individuals with severe neuromuscular disorders, enabling them to interact with external devices, such as computers, speech synthesizers, assistive technologies and artificial organs, to perform specific tasks [77]. In addition, BCI can enhance the healthy capabilities of individuals and demonstrate significant application prospects across various fields, including psychology, marketing, autonomous driving and aerospace. Currently, BCI is extensively utilized in various fields, such as scientific research, disease diagnosis, communication, disability assistance, industrial control, sleep analysis and more (Figure 14) [77]. It is anticipated that BCI will emerge as one of the key technologies driving societal advancement in the future.

The neural interface, also known as the neural electrode, is a fundamental component of BCI. This electrophysiological device facilitates interaction with neurons and is directly involved in the exchange of information between the brain and external devices. Neural interfaces can be categorized into implantable, semi-implantable and non-implantable based on their respective implantation methods. Clinical applications of implantable neural interfaces primarily rely on wire-based methods, which can lead to issues such as increased infection risk, limited placement flexibility and restricted patient mobility [78]. BCI offers a noninvasive alternative for measuring brain activity through electroencephalography (EEG). While this method avoids the costs and risks associated with surgical procedures, it suffers from limitations in acquisition potential and spatial resolution [79]. Consequently, there is a pressing need to enhance the efficiency and functionality of brain–computer interfaces (BMI) in recording, monitoring and stimulating bioelectronic signals.

**Figure 15.**
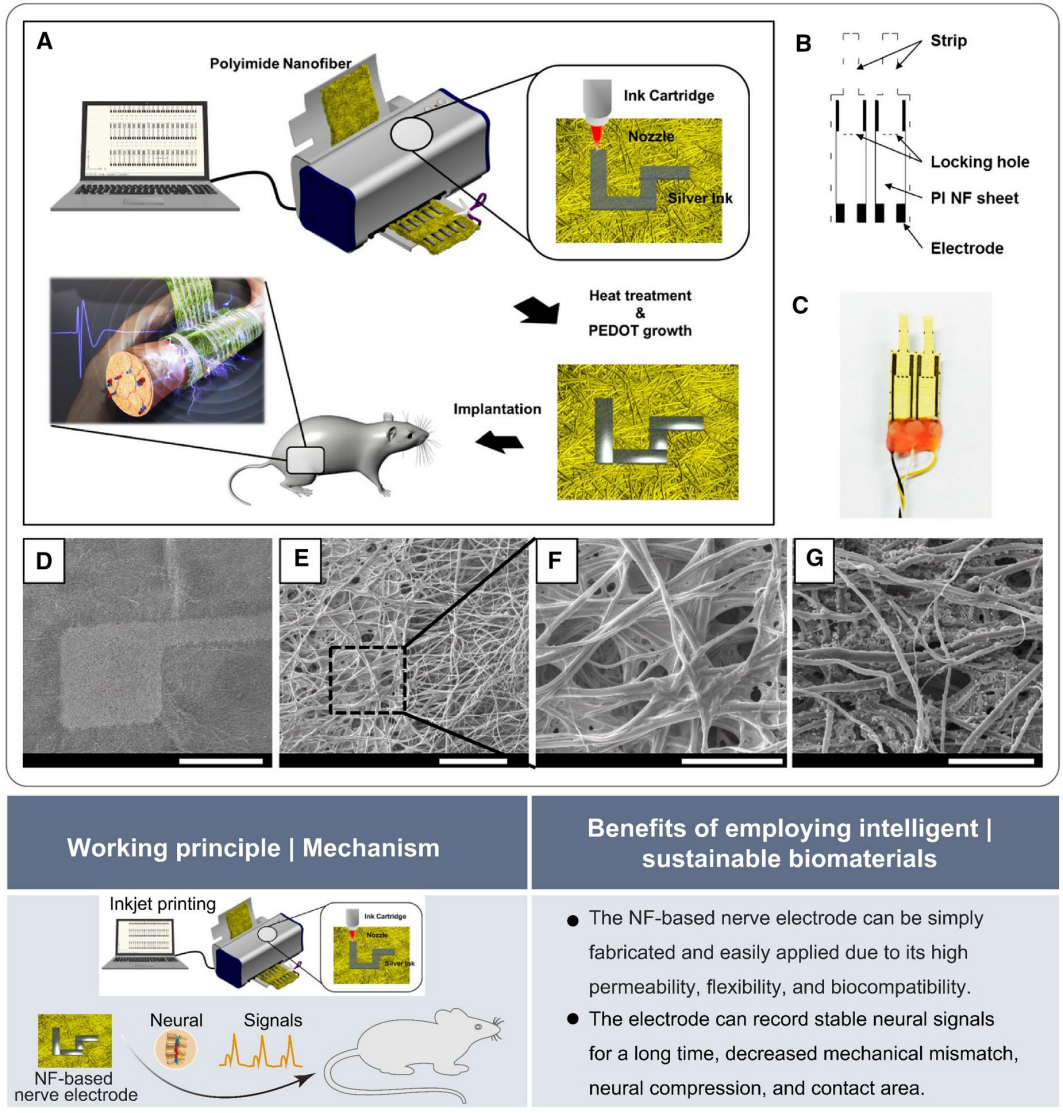
The application of biomaterials for neural electrodes. (**A**) Schematic illustration of the fabrication process utilizing an inkjet printing system. (**B**) Concept of preproduction design. (**C**) Image of the NF-based neural electrode. (**D–G**) SEM observed the microscopic morphology of electrodes. Reproduced from Ref. [81] with permission of American Chemical Society, © 2017.

Nanofibrous biomaterials have become prominent BCI application contenders, due to their large surface area, biocompatibility, biodegradability and customizable mechanical characteristics. These attributes enhance the efficiency of neural signal capture and transmission in BCI systems. In a recent study, a conductive neural interface material of PU, silk fibroin and functional multi-wall carbon nanotubes (fMWCNTs) was fabricated using electrospinning technology. This innovative material demonstrated significant potential in neural tissue engineering due to its exceptional mechanical properties, hydrophilicity and biodegradability [80]. In another study, a polyimide (PI) nanofiber (NF)-based neural electrode was reported, offering significant advantages in mitigating immune-mediated pathological tissue responses and enhancing long-term biocompatibility. These findings contribute to improved sustained biological connectivity and facilitate continuous monitoring of neural signals associated with the nervous system (Figure 15) [81]. Electrospun nanofibers are anticipated to attain a similar effect in the near future.

Future multi-task-based BCI, driven by advancements in sensor technology, analytical algorithms and AI, will be capable of collecting and analysing brain data over extended periods. These interfaces are anticipated to become integral components of daily life.

## Future directions

### Application of artificial intelligence

Artificial intelligence (AI) algorithms can extensively mine and analyse vast patient data. Integrating AI with intelligent biomaterials and sustainable biomaterials is poised to yield numerous innovative applications within the biomedical field. For instance, wearable devices fabricated from electrospun intelligent biomaterials can accurately detect physiological data such as heart rate, blood pressure and blood glucose levels. AI algorithms can rapidly analyse these large and complex datasets to provide early warnings about potential disease risks, while offering users personalized health management plans. In terms of intelligent drug delivery systems, AI has the potential to efficiently process extensive datasets from drug research and development. It can screen for promising drug targets and predict pharmacological activity, as well as potential side effects.

### Intelligence in the biomedical field

In the future, electrospun biomaterials in tissue engineering are expected to exhibit enhanced intelligence and precision. They will be capable of precisely detecting the minute changes during tissue repair, dynamically adapting to the surrounding microenvironment and accurately regulating the release timing and dosage of bioactive factors. This will effectively promote the orderly differentiation of cells and the reconstruction of tissues. In terms of drug delivery systems, the combination of electrospun intelligent biomaterials and drug delivery systems is expected to evolve toward ultra-miniaturization and a high degree of intelligence. These systems will achieve efficient drug loading and on-demand release by leveraging technologies like quantum dots and nanosensors [82, 83].

In disease monitoring and diagnosis, the sensitivity and specificity of sensors are expected to experience a substantial boost. They will accurately capture the subtle fluctuations in basic vital signs, such as heart rate, blood pressure and respiration, and monitor various biomarkers in real-time. For example, by tracking the concentration of specific substances in the blood, anomalies can be detected at the onset of the disease, even before clinical symptoms manifest, thereby realizing ultra-early warning. Simultaneously, in collaboration with wearable devices, BCI technology will be able to interpret brain activities more accurately and comprehensively. This will assist in assessing cognitive states, emotional changes and the risks of potential nervous system diseases, providing crucial evidence for the early diagnosis and intervention of such diseases.

In daily health management, wearable devices crafted from electrospun biomaterials will function as personal health guardians. They will continuously monitor physiological indicators, including heart rate, blood pressure, blood glucose and sleep quality. By analysing data with AI algorithms, they can issue early warnings of potential health risks. For example, when blood glucose levels are abnormal, the device can automatically release medications or provide personalized dietary and exercise recommendations, thereby enabling early prevention and intervention of diseases.

### Intelligent medical system

Biomaterials serve as the foundational basis for clinical applications, while AI offers support for clinical decision-making and the research and development of materials. The Internet of Things facilitates real-time data transmission and equipment interconnectivity. Together, these three components converge to form an intelligent medical system. In this system, biological materials are responsible for collecting and transmitting biological information. The Internet of Things relays this information to the cloud, and AI analyzes and processes the data to inform clinicians' decisions. This integration aims to deliver more efficient, accurate and personalized medical services. Establishing an intelligent medical system has catalyzed a shift in the medical model from traditional disease treatment toward a comprehensive cycle encompassing monitoring, diagnosis and treatment. Through real-time monitoring and data analysis, potential disease risks can be identified proactively, enabling early intervention. Personalized biological materials and advanced medical equipment enhance therapeutic outcomes during treatment processes. Furthermore, post-treatment recovery is supported through remote monitoring and rehabilitation guidance for effective patient health management.

Telemedicine is expected to undergo significant upgrades with the adoption of 5G and even more advanced communication technologies. Data collected by wearable devices can be transmitted to the medical cloud platform in milliseconds. Doctors, regardless of their geographical location, can conduct remote consultations and diagnoses for patients using high-definition video and virtual reality technologies. Based on accurate and real-time data, doctors can promptly adjust treatment plans.

To sum up, AI and big data will drive the intelligent transformation of the biomedical field. This will render treatment more efficient, safer and more precise, enabling the realization of patient-centered medical services. It will lead the biomedical field into a new era, bringing unprecedented benefits to human health.

## Conclusions

Electrospun biomaterials are increasingly being utilized in the field of biomedical, including human tissue repair (tissue engineering and regenerative medicine), medical monitoring (biosensors and BCIs), medical protection (personal protective equipment) and various medically related functions (drug delivery systems and wearable devices). This review explores the progress in electrospun biomaterials, including process innovations, operating principles and the impact of process variables. Also discusses the potential of electrospun intelligent and sustainable biomaterials in biomedical applications.

**Figure 16.**
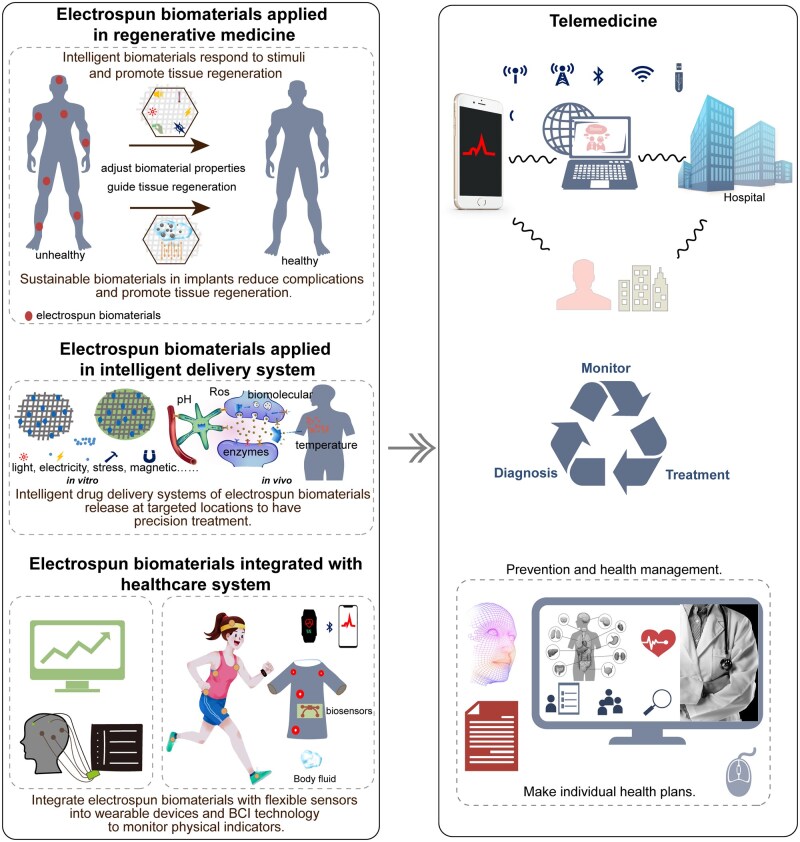
A schematic of future healthcare and the potential role of electrospun biomaterials. Electrospun biomaterials are increasingly utilized in regenerative medicine, where intelligent and sustainable materials adjust to changes within the human body to guide tissue regeneration. Intelligent drug delivery systems of electrospun biomaterials are released at targeted locations for precision treatment. Furthermore, electrospun materials can be integrated with BCI technology, biosensors and wearable devices, enabling enhanced monitoring of various physiological indicators. All collected data can be transmitted to hospitals via internet connections, facilitating remote monitoring, diagnosis and treatment, and improving disease prevention and management strategies.

The electrospun biomaterials offer research and innovation opportunities to realize the envisioned future of the healthcare system (Figure 16). Nowadays, multi-stimuli responsive intelligent scaffolds and sustainable scaffolds for tissue engineering can autonomously adjust mechanical properties, degradation rates and bioactive factors or drug release in response to different stages of tissue repair or variations in biomarkers or cellular signals, thereby guiding tissue regeneration. Intelligent drug delivery systems of electrospun biomaterials incorporate drugs into the fibers through physical mixing or chemical bonding. These systems are designed to respond to a variety of stimuli both *in vivo* (temperature, pH, enzymes, ROS, biomolecular concentration, etc) and *in vitro* (light, electricity, magnetic, stress, etc.) during their interaction with the human body, and then release at targeted locations to have precision treatment. Integrating electrospun materials with high-performance, flexible sensors into wearable devices and BCI technology enables the precise monitoring of vital signs, such as heart rate, blood pressure, respiration and brain activity. These devices transmit data over the internet, significantly supporting telemedicine by enabling remote diagnosis and treatment adjustments for physicians. Consequently, this could lead to more accurate disease predictions and personalized prevention strategies within intelligent medical systems, ultimately improving human well-being.
